# The harmful legacy of colonialism in natural hazard risk

**DOI:** 10.1038/s41467-022-34792-7

**Published:** 2022-11-14

**Authors:** Jazmin P. Scarlett

**Affiliations:** School of Environmental Sciences, University of East Anglia, Norwich, Norfolk NR4 7JT UK

**Keywords:** Natural hazards, Geography, Volcanology, Ethics

## Abstract

The colonial practices of geoscience have created long term vulnerabilities to natural hazards. In this comment the ongoing consequence are explored of colonialism as well as the actions that are needed to be taken to reduce natural hazard risk.

## Teaser

The legacies of the colonial practices of geoscience in creating long term vulnerabilities to natural hazards is often neglected in discussions of how to live with natural hazards today. Here, I explore the ongoing consequences of colonialism, along with actions that could improve future response to hazards by more fully acknowledging the impact of colonial pasts to improve our understanding of natural hazard risk.

The geosciences are rooted in colonial practices. Geosciences’ historical agenda was to aid the growth of colonial empires’ wealth, often at the expense of the local population via surveys and exploitation of landscapes^1,2^. The outcomes of these exploitative networks and practices have recently been through the lens of indigeneity and its influence on understanding and response to biodiversity^3,4^. Here, the focus is on natural hazard processes, which are an integral part of many landscapes around the world with varying degrees of risk for local populations. Colonialism and its harms are sometimes neglected in natural hazard research within geoscience. This comment considers the influence of colonial practices in several ways. They have acted to place local populations in locations of greater vulnerability to multiple hazards, with historical legacies resonating in the current day^5^. The relentless pursuit of economic resource has amplified vulnerabilities by the destruction of naturally available mitigations (such as forest cover), and finally the creation of knowledge around natural hazards is dominated by western understandings and practices. I explore ways in which diversifying understandings of natural hazards and acknowledging the colonial creation of risk can improve future preparedness and response.

## History and legacy of colonialism in geoscience

Although colonialism is often considered historical, the ramifications are still felt widely today. Geoscience is considered a ‘colonial science’, which has been shaped by social and political issues and agendas of colonial expansion^6^. The imperial agenda was to survey and map landscapes, waterscapes, and natural resources in colonised and occupied lands. Surveying and mapping of natural resources was part of the transfer of technical and specialist knowledge by European colonialism^7–9^. Many expeditions sought to document the landscape as a justification of who should live on and use the land, as well as using physical geography to designate the inferiority or superiority of human races that evolved in different landscapes^10^.

Historically, local indigenous populations were intellectually and socioeconomically exploited to gain access and extract natural resources that benefitted the colonial powers, usually in violation of indigenous peoples cultural understanding and significance of a site to them^11,12^. Sometimes the lack of understanding led to violence. In St. Vincent, in the Caribbean, the surveying of volcanic fertile soil in areas that were originally “reserved” for the indigenous peoples led to obtaining land through encroachment, voluntary sale and military force. This eventually resulted in two civil wars (The First and Second Carib War), and the forcible removal of the Garifuna to present-day Honduras. Subsequently, the land was used for some of the largest sugar plantations on the island, using enslaved African labour to produce sugar, molasses and rum to meet the demands of the British Empire in what we now know is in the high-risk zone of the volcano La Soufrière. The establishment of these plantations that turned into settlements’ post-emancipation that endure today, and this forced land settlement has led to greater risk from a variety of natural hazards, including volcanic eruptions, landslides, flooding and tropical storms^13^. Former colonised nations’ interactions with natural hazard processes within the exploited landscapes are part of the colonial legacy.

## Colonialism and natural hazards

With the overextraction of resources from the landscape, alongside this was the creation of a social hierarchy that marginalises particular social groups who are made vulnerable to natural hazards in a variety of ways, such as limited accessibility to resources to recover from natural hazard events and disasters. Often, marginalisation forces those who are not as socially, economically and/or politically mobile into high-risk areas such as floodplains, due to marginalised/low productive land, uneven development, economic barriers and natural hazard susceptibility^14–16^. This creates an environment where ‘resilience’ building has also been created under – and are thus a reproduction of — colonial and post-colonial strategies^17^.

Culturally embedded behaviours were developed over many cycles of experiencing natural hazards. However, the behaviours were undermined by colonists because they did not fit the ‘ideals’ of colonial behaviour, leading to financial, education and scientific knowledge shortcomings, which post-colonial societies struggle to overcome. These changed behaviours mean that local people can be more affected by natural hazards. In the case of Peru, colonialisation diminished pre-Columbian cultural adaptations to earthquakes. Urban planning concentrated indigenous peoples in new settlements for the purpose of social control and indoctrination, within structures using inappropriate construction materials that made them more vulnerable^18^.

The ideologies and philosophical framework of colonialism have been influencing our understanding of the environment and natural hazard phenomena, including the physical aspects of geohazards, without the inclusion of indigenous knowledges from people who lived in the landscapes experiencing natural hazard phenomena. For example, it was noted that despite deep Māori information and knowledge on earthquakes in Christchurch, Aotearoa New Zealand, their knowledge is eliminated from booklets and brochures^19^, however, there are increasing effects of Māori’s inclusion in urban recovery today to counteract this^20^.

Past and present colonialism has played a critical role in the ongoing anthropogenic climate crises and its resulting exacerbation of existing natural hazards. It is worth noting how the colonisation of indigenous lands in the United States and Australia has led to changes in vegetation type across landscapes and erasure of indigenous practices of controlled burning, which has led to an increase in both the number of and intensity of wildfires over the last decade^21^. As such, our ability to mitigate the natural hazards stemming from climate change is hindered by some of the same processes that initiated it. For geoscientists to effectively mitigate natural hazard processes today in a world where climate change is making these inequities worse, there needs to be more interrogation of the past – the connections between society and the changing of the environment.

Despite the examples given, it is important to acknowledge that the various ways colonialism impacts natural hazard risk varies from country to country, and there are many issues that are difficult to address.

## Actions for the future

Colonialism continues to block local geoscientists from researching natural hazard phenomena that they live with. This is done by removing agency in their own knowledge and understanding of natural hazard phenomena, the lack of resources to train homegrown geoscientists and to support the research of local geoscientists, who in some cases, must rely on the collaboration of overseas partners to access the funding. Western geoscience has historically excluded different types of knowledge. But these excluded perspectives and voices would be just as, or even more so, valuable to live in a landscape that is continuously becoming hazardous in light of the effects of a changing climate and an increasing population.

There are multiple approaches to address the colonial legacies in geoscience, such as through teaching, research, strong collaborations. One would be to implement teaching the next generations of geoscientists to break the cycle - to have courses, lectures and resources like *Geocontext*^10^. *Geocontext* provides resources that integrate topics on racism, colonialism, imperialism, environmental damage and exploitation of natural resources into subjects commonly taught within geoscience programs. Whilst these resources are aimed for the US curriculum, inclusion of *Geocontext* studies can also be supported more broadly, especially since many geoscientists work overseas. This is starting to be addressed in the UK by acknowledging the lack of diversity in geoscience in scientific special interest groups, research and approaches to decolonising the geology curriculum^22–24^. This also relates to the field of geoethics that seeks to utilise a framework to address not only how geoscience researchers are responsible for sustainable interaction with earth systems and in the research that they conduct^25^. However, geoethics as a field and implementation into wider geoscience curriculum and research practice is slow, although progress has been made in integrating geoethics into the curriculum has been made in physical geography^26^.

An additional approach is the acknowledgement that the historical development of geoscience and the people that conducted it, should no longer be separated and importantly, that it is okay to change. For example, the University of Glasgow recently acknowledged and renamed a building originally named after the geologist John Walter Gregory, who documented the East African Rift (also known as the Gregory Rift), after learning he supported white supremacy and called for racial segregation^27^. In depth work can be done not only on Gregory’s contribution to geology, but also demonstrating the social and political contexts that shaped Gregory’s views.

Another option is to recognise and avoid parachute science (the practice of side-lining local researchers on field studies conducted in their own countries^28^) by working meaningfully with local knowledge. The ideologies of colonialism ran deep and touched all aspects of society that are both tangible and intangible, and therefore require the same level of scrutiny into understanding how people have lived and continue to live with natural hazards. This is not to say that geoscientists must now also be social scientists and historians, but instead develop equitable partnerships from research inception and design, especially in natural hazards research – involve local social scientists and historians who have local knowledge in the creation of hazard and risk assessments.

There is also the opportunity to learn from indigenous and cultural knowledge in living with natural hazards. For example, there is an interconnectivity among human, non-human and the metaphysical in making sense of disasters in Zamboanga Peninsula, Southern Philippines^29^. While indigenous knowledge of earthquakes in Indonesia has led to adaptation of house construction that have endured for centuries^30^. These examples and more, have seen a mixture of success from research into implementation however, if to be incorporated into the postcolonial society, they will require a large cultural shift and retrofitting of infrastructure that may not be possible for some countries at present.

It may be easy to dismiss colonialism as something that ‘happened in the past’. But for many people living in once occupied locations, it is generational trauma that cannot be so easily forgotten, as there is a continued perpetuation of colonialism. Being complicit to these knowledge systems touched by white supremacy and imperialism is violence. There is no shame in acknowledging the tainted roots of our disciplines and there should be celebration in wanting to do and be better, as that is a benefit for all, not just the privileged few.
